# Hadamard Kernel SVM with applications for breast cancer outcome predictions

**DOI:** 10.1186/s12918-017-0514-1

**Published:** 2017-12-21

**Authors:** Hao Jiang, Wai-Ki Ching, Wai-Shun Cheung, Wenpin Hou, Hong Yin

**Affiliations:** 1 Department of Mathematics, School of Information, Renmin University of China, No.59 Zhong Guan Cun Avenue, Hai Dian District, Beijing, 100872 China; 2 Department of Mathematics, The University of Hong Kong, Pokfulam Road, Hong Kong, Hong Kong

**Keywords:** SVM, Hadamard Kernel, Breast Cancer

## Abstract

**Background:**

Breast cancer is one of the leading causes of deaths for women. It is of great necessity to develop effective methods for breast cancer detection and diagnosis. Recent studies have focused on gene-based signatures for outcome predictions. Kernel SVM for its discriminative power in dealing with small sample pattern recognition problems has attracted a lot attention. But how to select or construct an appropriate kernel for a specified problem still needs further investigation.

**Results:**

Here we propose a novel kernel (Hadamard Kernel) in conjunction with Support Vector Machines (SVMs) to address the problem of breast cancer outcome prediction using gene expression data. Hadamard Kernel outperform the classical kernels and correlation kernel in terms of Area under the ROC Curve (AUC) values where a number of real-world data sets are adopted to test the performance of different methods.

**Conclusions:**

Hadamard Kernel SVM is effective for breast cancer predictions, either in terms of prognosis or diagnosis. It may benefit patients by guiding therapeutic options. Apart from that, it would be a valuable addition to the current SVM kernel families. We hope it will contribute to the wider biology and related communities.

**Electronic supplementary material:**

The online version of this article (doi:10.1186/s12918-017-0514-1) contains supplementary material, which is available to authorized users.

## Background

It is known that 13% of deaths all over the world are caused by cancer [1]. For women, breast cancer is a leading cause of deaths world-wide. In the U.S. alone, it is estimated that 246,660 new patients will be diagnosed with breast cancer, and 40,450 deaths associated with malignancy are estimated [2]. Early detection and identification of breast cancer is necessary for reducing the side-effects of the disease. On the other hand, cancer prognosis can assist in designing treatment protocol which is also of great importance. Cancer prognosis can be interpreted as estimating survival probability within a certain period of time. A 10-year prognosis of 60% represents the probability of surviving 10 years after surgery or diagnosis is 60%. Here we formulate the prognosis problem as a classification one where label information can be retrieved from the survival information beyond the prognosis period. For example, patients who died before the considered prognosis period are labeled negative and vice versa.

In cancer research, cDNA Microarrays and high density oligonucleotide chips are increasingly used and in the meantime they raise numerous excellent and challenging research problems in fields. By monitoring expression levels in cells for tens of thousands of genes simultaneously, microarray experiments may lead to a better understanding of the molecular variations among tumors and hence to a more informative classification [3]. Over the last few years, substantial efforts [4–7] have been made on gene expression profile based classifiers for predicting patient outcomes in breast cancer.

Maglogiannis et al. [8] proposed Support Vector Machines (SVMs) based classifier for the prognosis and diagnosis of breast cancer disease. It was compared with Bayesian classifiers and Artificial Neural Networks (ANNs). Delen et al. [9] compared three algorithms for predicting breast cancer survivability where they used SEER data for evaluation. Endo et al. [10] proposed optimal model for 5-year prognosis of breast cancer. They compared seven algorithms (Logistic Regression model, ANN, Naive Bayes, Bayes Net, Decision Trees with Naive Bayes, Decision Trees (ID3) and Decision Trees (J48)) on SEER data and results show that decision tree J48 showed the highest sensitivity, ANN had the highest specificity. We note that the data used for model comparisons in [9, 10] is very large in samples (over 30,000) but relatively small in attributes. When the data sets involved small number of samples, SVM based algorithms can usually outperform other considered algorithms. Vikas et al. [11] compared Naive Bayes, SVM-RBF kernel, RBF neural networks, Decision Trees (J48) and Classification And Regression Tree (CART) to find the best classifier for the breast cancer data sets. Experimental on 286 samples show that SVM-RBF kernel is more accurate. Aruna et al. [12] compared SVM, Decision Tree, and RBF Neural Networks in prediction of Wisconsin Breast Cancer Dataset (there are 699 samples). Results show that SVM-RBF kernel is the best among the considered methods. Asri et al. [13] compared SVM, Decision Tree (C4.5), Naive Bayes, K-Nearest Neighbors (KNN) on the Wisconsin Breast Cancer Datasets to assess the efficiency and effectiveness of algorithms. Experimental results show that SVM yields the highest accuracy.

In the current perspective, SVM demonstrates as a benchmark for various disciplines in particular for dealing with small sample problems. The effectiveness of SVMs depends on the choice of kernels. In [14], we proposed a novel kernel based on correlation matrix for cancer diagnosis purpose. Experiments on 5 real-world cancer data sets with gene expression profiles showed that correlation based kernel outperformed other classical kernels.

In this paper, we propose a parsimonious kernel named Hadamard Kernel for breast cancer outcome predictions. The remainder of this paper is structured as follows. In “Method” section, we propose the parsimonious positive semi-definite kernel. Theoretical proof on the positive semi-definite property of the kernel is provided. In “Results” section, publicly available data sets are utilized to check the performance of the proposed method. Finally, concluding remarks are given in “Conclusions” section.

## Method

- **Preliminaries**
  The basic SVM considers binary classification problem through building an appropriate model representing data points, mapping them so as to best separate different categories. In a formal setting, if we assume a data set of *n* data instances with corresponding class annotations:
  $$\left\{\left(\mathbf{x}_{1},y_{1}\right),\cdots,\left(\mathbf{x}_{n},y_{n}\right)\right\} $$
  where **x**_*i*_∈**R** ^*p*^,*y*_*i*_∈{−1,1}. SVM constructs a hyperplane to ensure good separation having largest distance from it to the nearest data points in each class category [15]. The optimization problem can be formulated as follows:
  1 $$ \left\{ \begin{array}{l} \text{Minimize} \ \frac{1}{2}{\|\mathbf{w}\|}^{2} \\ \text{subject to} \ y_{i}\left(\mathbf{w} \cdot \mathbf{x}_{i} -b\right)\geq 1 \\ \text{for any} \ i \in \{1,2,\ldots,n\} \end{array} \right.  $$
  The dual form of the primal optimization problem is given by:
  2 $$ \left\{ \begin{array}{l} \text{Maximize} \sum_{i=1}^{n}\alpha_{i} -\frac{1}{2}{\alpha}^{T}\mathbf{H} {\alpha} \\ \text{subject to} \ \alpha_{i} \geq 0 \\ \text{for any} \ i \in \{1,2,\ldots,n\} \\ \sum_{i=1}^{n}\alpha_{i} y_{i}=0 \end{array} \right.  $$
  where *α*=[*α*_1_,*α*_2_,…,*α*_*n*_],
  $$\mathbf{H}=\left(\begin{array}{cccc} y_{1}^{2}\mathbf{x}_{1}^{T}\mathbf{x}_{1} & y_{1}y_{2}\mathbf{x}_{1}^{T}\mathbf{x}_{2} & \ldots & y_{1}y_{n}\mathbf{x}_{1}^{T}\mathbf{x}_{n} \\ y_{2}y_{1}\mathbf{x}_{2}^{T}\mathbf{x}_{1} & y_{2}y_{3}\mathbf{x}_{2}^{T}\mathbf{x}_{3} & \ldots & y_{2}y_{n}\mathbf{x}_{2}^{T}\mathbf{x}_{n} \\ \vdots & \vdots & \ddots & \vdots \\ y_{n}y_{1}\mathbf{x}_{n}^{T}\mathbf{x}_{1} & \ldots & \ldots & y_{n}^{2}\mathbf{x}_{n}^{T}\mathbf{x}_{n} \\ \end{array} \right) $$
  When the data sets are nonlinearly separable, one can construct a nonlinear mapping for input vectors into feature space of higher dimensionality [16]. Different from previous setting based on inner product of input vectors, kernel matrix is constructed in terms of similarity measure through pairwise comparisons. Given *n* data instances **X**={**x**_1_,**x**_2_,…,**x**_*n*_}, kernel matrix *K* is a *n*×*n* matrix which is symmetric, i.e.,
  $$K(\mathbf{x},\mathbf{x}') = K(\mathbf{x}',\mathbf{x}) $$
  for any **x**,**x** ^′^∈**X**.
  There are a number of popular kernels, the most straightforward one is:
  Linear Kernel.
  $$K(\mathbf{x},\mathbf{x}') = \mathbf{x}^{T}\mathbf{x}', $$
  which is an inner product of **x** and **x** ^′^ in **R** ^*p*^.
  Another popularly used kernel matrix is polynomial kernel that is expressed as
  $$K(\mathbf{x},\mathbf{x}') = \left(\mathbf{x}^{T}\mathbf{x}'+1\right)^{d}, $$
  Gaussian Radial Basis Function (RBF) kernel is defined as
  $$K(\mathbf{x},\mathbf{x}')=\text{exp}\left(-{d\|\mathbf{x}-\mathbf{x}'\|^{2}}\right) $$
  where *d* is parameter. If the distance between **x** and **x** ^′^ is small, the kernel value would be large; on the contrary, if **x** is far away from *x* ^′^ in terms of Euclidean distance, the kernel value would be small. Hence this kernel provides a similarity measure between data points.
- **Hadamard Kernel**
  Kernel trick is useful in the sense that there is no need to calculate *ϕ*(**x**) explicitly as long as constructing appropriate kernel matrix. The Positive Semi-Definite (PSD) property [17] of a kernel matrix is required to ensure the existence of a Reproducing Kernel Hilbert Space (RKHS) where a convex optimization formulation can be deduced to yield an optimal solution.
  We propose Hadamard Kernel in this way:
  $$K_{\alpha}\left(\mathbf{x}_{i},\mathbf{x}_{j}\right)=\sum_{k=1}^{p}\frac{|x_{ik}|^{\alpha} |x_{jk}|^{\alpha}}{2\left(|x_{ik}|^{\alpha}+|x_{jk}|^{\alpha}\right)}, i,j = 1,2,\ldots, n. $$
  Here *α*≠0 is a flexible parameter within the kernel matrix. For some *k*, if *x*_*ik*_=0, then $\frac {|x_{ik}|^{\alpha } |x_{jk}|^{\alpha }}{(|x_{ik}|^{\alpha }+|x_{jk}|^{\alpha })}$ is defined as 0.

### **Theorem**

Kernel *K*_*α*_ is positive semi-definite for all data matrix **X**.

The proposed Hadamard Kernel with varying parameter *α* constitute to a broad range of kernel families which can fit all kinds of data matrix if the theorem holds.

Before we prove the theorem, let’s first consider the following kernel:

$$K_{0}\left(\mathbf{x}_{i},\mathbf{x}_{j}\right)=\sum_{k=1}^{p}\frac{x_{ik}x_{jk}}{2\left(x_{ik}+x_{jk}\right)}, \ i,j = 1,2,\ldots, n. $$

For some certain *k*, if *x*_*ik*_=0, then $\frac {x_{ik}+x_{jk}}{x_{ik}x_{jk}}$ is defined as 0.

This kernel is not generally positive semi-definite. Let’s consider the following example.

### **Example**

Assume **x**_1_=[ 1,1⋯1]^*T*^,**x**_2_=[ −1/2,−1/2⋯−1/2]^*T*^, then the kernel matrix has the following structure:

$$\left(\begin{array}{ccc} n & -2n & \cdots \\ -2n & -n/2 & \vdots \\ \vdots & \cdots & \ddots \\ \end{array} \right) $$

$$\begin{aligned} If\ \vec{x}&=[\!1,1,0,\cdots,0]^{T}, \text{we have}\\ \mathbf{x}^{T}K_{0}\mathbf{x}&=-7n/2<0 \quad \text{when} \quad n\geq 1\\ If\ \mathbf{x}&=[\!-1,0,\cdots,0]^{T}, \text{we have}\\ \mathbf{x}^{T}K_{0}\mathbf{x}&=n>0 \quad \text{when} \quad n\geq 1\\ \end{aligned} $$

∘

However, in our particular case, where all the gene expression values are positive valued, the kernel here is positive semi-definite. We give the proof in the subsequent statement.

### **Theorem**

Kernel *K*_0_ is positive semi-definite when that data matrix **X** is positive valued.

### *Proof*

For a positive matrix *A*=(*a*_*ij*_), we define the Hadamard inverse of *A* by $ A^{\circ (-1)}=\left (\frac 1{a_{ij}}\right). $ First proved by Bapat [18] and reformulated by Reams [19], we have the following proposition. □

### **Proposition**

If *A* is a positive symmetric matrix with only one positive eigenvalue, then *A* ^∘(−1)^ is positive semi-definite.

Let $e=(1, \ldots, 1), \mathbf {X}=\left [\begin {array}{c} \mathbf {x}_{1} \\ \mathbf {x}_{2} \\ \vdots \\ \mathbf {x}_{n} \end {array}\right ] =[ \mathbf {w}_{1}, \mathbf {w}_{2}, \ldots, \mathbf {w}_{p}]$, and define $V_{\mathbf {X}}(i,j)=\left (\sum _{r=1}^{p} \frac {1}{\mathbf {X}_{ir}+\mathbf {X}_{jr}}\right)$. We therefore have

$$K=\frac12V_{\mathbf{X}^{\circ(-1)}}. $$

To show that *K* is always positive semi-definite is equivalent to show that *V*_**X**_ is always positive semi-definite.

To show that *V*_**X**_ is positive semi-definite, we only need to show that $\phantom {\dot {i}\!}V_{\mathbf {x}_{i}e}$ is positive semi-definite for any positive column vector **w**_*i*_ as we have

$$V_{\mathbf{X}}=V_{\mathbf{w}_{1}e}+\cdots V_{\mathbf{w}_{p}e}. $$

Suppose **w**_*i*_=(*x*_1*i*_,…,*x*_*ni*_)^*T*^, then we have $V_{\mathbf {w}_{i}e}=\left (\mathbf {w}_{i}e+\left (\mathbf {w}_{i}e\right)^{T}\right)^{\circ (-1)}$.

Note that **w**_*i*_ *e*+(**w**_*i*_ *e*)^*T*^ is a positive symmetric matrix of rank 2 and it is not positive semi-definite (the determinant of any principal 2×2 submatrix is negative), hence it has exactly one positive eigenvalue. Therefore by the result of Reams, $\phantom {\dot {i}\!}V_{\mathbf {x}_{i}e}$ is positive semi-definite.

We can generalize the result to any non-negative matrix **X** as well.

### **Theorem**

Kernel *K*_0_ is positive semi-definite when data matrix **X** is non-negative.

### *Proof*

For *x*,*y*≥0, we define the binary operation

$$x\cdot y= \begin{cases}\frac{x+y}{xy}=\frac{1}{x^{-1}+y^{-1}} & \text{if }xy \ne 0\\ 0 \quad \text{if}\ xy=0\end{cases}. $$

Here $K=U_{\mathbf {X}}=\frac {1}{2}\left (\sum _{r=1}^{p}\mathbf {X}_{ir}\cdot \mathbf {X}_{jr})\right)$.

Then $\phantom {\dot {i}\!}U_{\mathbf {X}}=U_{\mathbf {w}_{1}e}+\cdots U_{\mathbf {w}_{p}e}$. To show that *U*_*X*_ is positive semi-definite, we only need to show that *U*_**w***e*_ is positive semi-definite for any nonnegative column vector **w**.

Suppose **w** has zero entries. Without loss of generality, write **w**=(**y** **,** **0**)^*T*^ where **y**>0, then $U_{\mathbf {w}e}=\begin {pmatrix}U_{\mathbf {y}e}&0\\0&0\end {pmatrix}$ which is positive semi-definite if and only if *U*_**y***e*_ is positive semi-definite. Hence it suffices to show that *U*_**y***e*_ is positive semi-definite for any positive column vector **y**.

Take **y** ^′^ to be the Hadamard inverse of **y**, then *U*_**y***e*_=(**y** ^′^ *e*+(**y** ^′^ *e*)^*T*^)^∘(−1)^. Note that **y** ^′^ *e*+(**y** ^′^ *e*)^*T*^ is a positive symmetric matrix of rank 2 and it is not positive semi-definite (the determinant of any principal 2×2 submatrix is negative), hence it has exactly one positive eigenvalue. Therefore by the result of Reams [19], *U*_**w***e*_ is positive semi-definite. □

We now proceed to prove the first theorem.

### *Proof*

For any data sample within matrix **X**, we can generate a corresponding new matrix **X**_*α*_ by

$$\mathbf{X}_{\alpha}(i,j)=|x_{ij}|^{\alpha}. $$

Then according to the previous theorem, we can show the validity of the theorem. □

- **Models for comparison**
  In this paper, we consider breast cancer outcome predictions based on high dimensional gene expression profiles. Hence the number of samples is relatively small. In the literature it is shown that SVM is statistically better than other machine learning algorithms. We therefore confine our research in the framework of SVMs and exclude other algorithms from our scope of research. Other kernels for a comparison are listed below.
- SVM Linear Kernel
  $$K(\mathbf{x},\mathbf{x}') = \mathbf{x}^{T}\mathbf{x}', $$
- SVM Quadratic Kernel
  $$K(\mathbf{x},\mathbf{x}') = \left(\mathbf{x}^{T}\mathbf{x}'+1\right)^{2}, $$
- SVM RBF Kernel
  $$K(\mathbf{x},\mathbf{x}')=\text{exp}\left(-{\frac{\|\mathbf{x}-\mathbf{x}'\|^{2}}{\sigma^{2}}}\right) $$
- SVM Correlation Kernel
  This kernel construction can be decomposed into three steps.
  1. Based on the correlation matrix, we first construct a preliminary kernel.
  $$ K_{CB} = 1 - e^{-{\text{corr}}(\mathbf{X})} $$
  2. We do eigenvalue decomposition for the matrix *K*_*CB*_ where *V* is the matrix composed of eigenvectors, *P* is the diagonal matrix where diagonal entries are eigenvalues.
  $$K_{CB}=V^{T}PV $$
  3. Denoising strategy. If we denote
  $$ P = \left(\begin{array}{cccc} p_{1} & 0 & \cdots & 0 \\ 0 & p_{2} & \cdots & 0 \\ \vdots& \vdots & \ddots & \vdots \\ 0 & 0 & \cdots & p_{n} \\ \end{array} \right). $$
  The denoising strategy is to transform the diagonal matrix *P* to another diagonal matrix $\tilde {P}$,
  $$ \tilde{P} = \left(\begin{array}{cccc} \tilde{p}_{1} & 0 & \cdots & 0 \\ 0 & \tilde{p}_{2} & \cdots & 0 \\ \vdots& \vdots & \ddots & \vdots \\ 0 & 0 & \cdots & \tilde{p}_{n} \\ \end{array} \right) $$
  where
  $$\tilde{p}_{i} = \left\{ \begin{array}{ll} 0, & \text{\(p_{i} < 0\);} \\ p_{i}, & \text{\(p_{i} \geq 0\).} \end{array} \right., \ i = 1, 2, \ldots, n. $$
  Finally, the kernel matrix becomes
  $$ K_{DCB}=V^{T}\tilde{P}V. $$

## Results

### Materials

We obtained a number of real-world data sets from National Center for Biotechnology Information [20]. The first data set is derived from a N-methyl-N-nitrosourea-induced breast cancer model. It has 35 samples in total, of which 11 are normal. The number of attributes used to describe a sample is 15923. Expression profiles were obtained through Affymetrix Rat Expression 230A Array. The annotation ID for this data set is GSE1872.

Estrogen Receptor-Positive (ER+) and ER- breast cancers tend to show different patterns of metastasis. In this data set where the access number is GSE32394, glycan structure analyse by Custom Affymetrix Glyco v4 GeneChip was conducted to compare the two types of breast cancer. There are 19 samples in total, of which 9 are ER+, the number of attributes is 1259.

The third data set is used to differentiate non-invasive breast cancer and invasive breast cancer, the access number is GSE59246. mRNA, miRNA and DNA copy number profiles are generated to measure the expression of different samples. Arrays consist of 3 normal controls, 46 ductal carcinoma in situ lesions, and 56 small invasive breast cancers. We discard the 3 normal controls, so we have 102 samples in total. In this data set, the number of attributes is 62976.

Studies show that circulating miRNAs have the potential to become biomarkers. This data set involves 78 samples in total, 1205 circulating miRNAs for measurements. 26 of the 78 samples are negative. Identification number for this data set in NCBI is GSE59993.

One more data set is related to breast cancer prognosis, GSE25055 is the identification number. A total number of 310 breast cancer patients is involved. The number of attributes is 22283. This study is conducted with Affymetrix Human Genome U133A Array. It is a neoadjuvant study of HER2-negative breast cancer cases treated with taxane-anthracycline chemotherapy pre-operatively and endocrine therapy if ER-positive. Response was assessed at the end of neoadjuvant treatment. Using 5 years as a cutoff, we conduct the outcome prediction.

The last data set contains 60 patients with ER-positive primary breast cancer and treated with tamoxifen monotherapy for 5 years [21], the identification number in NCBI is GSE1379. This study was conducted using expression profiling by array, with the number of attributes 22575. We build models to predict the 5-year recurrence outcome for the considered patients. There were 28 patients who showed recurrence symptoms.

### Performance evaluation

#### 5-fold cross validation

Cross validation is a standard way to evaluate the supervised learning model. The *k*-fold cross validation is performed as follows: first of all, the training data set $\mathcal {M}$ is randomly divided into *k* subsets $\mathcal {M}_{1},\cdots,\mathcal {M}_{k}$ of approximately equal size. The prediction model is trained on *k*−1 subsets and the remaining subset is treated as the test set. Repeating this process *k* times such that each subset is tested once, all the prediction results are recorded for the computation of prediction accuracy. In our case, we conduct 5-fold cross validation for model evaluations.

#### Area under the receiver operating characteristic (ROC) curve

In the context of classification, suppose the two true classes are *P* (positive) and *N* (negative), while the predicted positive and negative classes are *P* ^′^ and *N* ^′^, respectively. This is illustrated by Table 1 below where ROC Curve is a graphical plot of False Positive Rate (FPR) vs. True Positive Rate (TPR) as *x* and *y* axes, respectively, for a binary classifier system as its discrimination threshold is varied. FPR and TPR are defined as follow:

$$\text{FPR} = \frac{fp}{tn+fp} \quad \text{and} \quad \text{TPR} = \frac{tp}{tp+fn}. $$

TPR determines a classifier performance on classifying positive instances correctly among all positive samples available during the test, while FPR defines how many incorrect positive results occur among all negative samples available during the test. Each prediction result represents one point on the ROC curve. The best possible prediction method would yield a point in the upper left corner or coordinate (0,1) of the ROC space, representing no false negatives and no false positives.

**Table 1:** Definitions for True/False Positive/Negatives

Result		
True	*P* ^′^	*N* ^′^
*P*	True positive (*tp*)	False negative (*fn*)
*N*	False positive (*fp*)	True negative (*tn*)

The area under the ROC curve (AUC) [22, 23] is a widely adopted statistics for assessing the discriminatory capacity of models. It can be interpreted as a measure of aggregated classification performance, and also the tradeoff between specificity and sensitivity [24].

### Experimental results

In this section, we will show the performance of the Hadamard Kernel in conjunction with SVM and the other 4 kernels for breast cancer outcome predictions as tested on the five data sets. We employed the AUC measured by 5-fold cross-validation run 10 times to evaluate the performance. All the experiments are conducted using Matlab R2012 under Window 7 Operations System.

In RBF kernel, we have to specify the parameter *σ* before model training. Therefore, we initially conduct 10 time 5-fold cross validation solely on RBF kernel with *σ*∈{10^−2^,10^−1^,1,10,100,1000}. Averaged AUC Values with corresponding standard deviations are shown in Table 2. For example, in GSE1872 data set, the performance of RBF kernel is not sensitive to different values of *σ*. The best *σ* of RBF kernel for GSE32394 and GSE59246 breast cancer prediction is 1000 whereas the best *σ* for GSE59993 is 10. Particular case can also arise when RBF kernel is insensitive to values of *σ*. We can draw the conclusion that there is no optimal *σ* for all the considered data sets and different data sets may have different best *σ*.

**Table 2:** Averaged AUC values for determining optimal *σ* in RBF kernel

*σ*						
Datasets	*σ*=0.01	*σ*=0.1	*σ*=1	*σ*=10	*σ*=100	*σ*=1000
GSE1872	0.2379 ± 0.0538	0.2379 ± 0.0538	0.2379 ± 0.0538	0.2379 ± 0.0538	0.2379 ± 0.0538	0.2379 ± 0.0538
GSE32394	0.1811 ± 0.0707	0.1811 ± 0.0707	0.2044 ± 0.0845	0.6767 ± 0.1125	0.9456 ± 0.0133	**0.9456 ± 0.0122**
GSE59246	0.4408 ± 0.0446	0.4408 ± 0.0446	0.4408 ± 0.0446	0.4408 ± 0.0446	0.8424 ± 0.0379	**0.8658 ± 0.0110**
GSE59993	0.3542 ± 0.0283	0.3542 ± 0.0283	0.4305 ± 0.0355	**0.8392 ± 0.0235**	0.6937 ± 0.0340	0.6940 ± 0.0342
GSE25055	0.3651 ± 0.0182	0.3651 ± 0.0182	0.3651 ± 0.0182	0.3651 ± 0.0182	**0.8092 ± 0.0156**	0.7259 ± 0.0127
GSE1379	0.3952 ± 0.0478	0.3952 ± 0.0478	0.3982 ± 0.0468	0.3970 ± 0.0468	**0.6712 ± 0.0294**	0.6276 ± 0.0374

The bold face represents best performance detected for different considered *σ*

For hadamard kernel, we would like to see the performance of Hadamard Kernel in relation with parameter *α*. Figures S1 to S6 (attached in Additional file 1) record the performance of Hadamard Kernel in relation with parameter *α* from (0,5) with step size 0.1. Optimal *α* in Hadamard Kernel varies in different data sets. For example, one can see a steady decrement in performance when *α*>1.3 in GSE1872 and when *α*>2.8 in GSE59246. There is no obvious pattern detected in Additional file 1: Figure S2 in GSE32394, the performance is unstable with respect to *α*. But we can see a tendency of decrement in an overall manner. For GSE59993, the performance is firstly increasing, achieving the best for *α*=0.5. The performance is then decreasing steadily. In GSE25055, the performance of hadamard kernel stays in a stable range when *α*<2.8, it then decreases drastically. For GSE1379, the performance of hadamard kernel gradually increases when *α*>2. It can be seen that different datasets may fit for different best *α* in Hadamard Kerne, the optimal *α* determination becomes an interesting problem.

Figures S7 to S12 (attached in Additional file 1) depict the AUC values of the 5 considered methods in each 5-fold cross validations. Dark blue refers to Hadamard Kernel, Linear Kernel is marked in blue, green represents Quadratic Kernel, and orange stands for RBF Kernel, brown stands for Correlation Kernel. In the *x*-axis, 1 represents the first 5-fold cross-validation. The corresponding values in y axis are the AUC values for the considered 5 methods. For example, in Additional file 1: Figure S7 for GSE1872, the best performance is shown in Hadamard Kernel and Correlation Kernel in the first round, achieving 100% in accuracy. The performances of Linear Kernel, RBF Kernel and Quadratic Kernel are not satisfactory. RBF Kernel shows the worst performance, the AUC values are below 30%. Similar patterns can be detected in the remaining 9 round 5-fold cross-validations. In summary, Hadamard Kernel and Correlation Kernel show the best performance regarding the 10 runs 5-fold cross-validations.

Additional file 1: Figure S8 shows the performance of different models for data set GSE32394. The best performance is shown in Hadamard Kernel, it is slightly better than Linear Kernel. RBF Kernel and Correlation Kernel show comparable performance, and the worst performance is shown in the Quadratic Kernel.

Additional file 1: Figure S9 depicts the result for GSE59246 breast cancer outcome prediction. Hadamard Kernel still demonstrates the best performance, the second best performance is shown in Linear Kernel. Overall, RBF Kernel is better than Correlation Kernel. They rank the third the fourth place this time. Quadratic Kernel can only get 50% in AUC values on average.

In GSE59993, Hadamard Kernel is better than the other 4 kernels as shown in Additional file 1: Figure S10. RBF Kernel shows the second best in this context. Linear Kernel ranks the third place and Quadratic Kernel shows the worst performance.

Additional file 1: Figure S11 shows the result for GSE25055 breast cancer outcome prediction. GSE25055 is a data set related to breast cancer prognosis. We formulate the problem into a classification one by labeling patients who survive within 5 years after diagnosis as positive classes. Hadamard Kernel and Linear Kernel reach the top places, yielding around 84% on average in AUC values. The performance of RBF kernel is also acceptable, achieving around 81% in Averaged AUC Values.

Additional file 1: Figure S12 reports the result for GSE1379, a data set related to ER-Positive breast cancer recurrence status prediction. It can be clearly shown that hadamard kernel shows the best performance, RBF kernel ranks the second best, and Quadratic kernel ranks the worst.

Table 3 illustrates the average AUC value over the 10 runs with standard deviations. The best performance is marked in bold face. It is clear to see that on average Hadamard Kernel shows the best performance on all the considered data sets.

**Table 3:** Averaged AUC values for comparison of different methods

Methods					
Datasets	Linear kernel	Quadratic kernel	RBF kernel	Hadamard kernel	Correlation kernel
GSE1872	0.3788 ± 0.1019	0.3686 ± 0.1136	0.2117 ± 0.0584	**1.000 ± 0.000**	0.9989 ± 0.0018
GSE32394	0.9456 ± 0.0312	0.5544 ± 0.1248	0.9344 ± 0.0254	**0.9589 ± 0.0166**	0.9233 ± 0.0294
GSE59246	0.8977 ± 0.0172	0.5386 ± 0.0579	0.8431 ± 0.0379	**0.9022 ± 0.0145**	0.8562 ± 0.0113
GSE59993	0.8283 ± 0.0226	0.5935 ± 0.0694	0.8347 ± 0.0182	**0.8855 ± 0.0088**	0.7869 ± 0.0144
GSE25055	0.8575 ± 0.0182	0.4743 ± 0.0393	0.8196 ± 0.0203	**0.8653 ± 0.0171**	0.7654 ± 0.0152
GSE1379	0.6205 ± 0.0481	0.5237 ± 0.0701	0.6743 ± 0.0427	**0.7300 ± 0.0375**	0.6419 ± 0.0453

The bold face represents the best performance detected for different compared methods

To sum up, Hadamard Kernel is effective and robust in predicting breast cancer outcomes. There is no dominant algorithm for the other 4 considered kernels. Quadratic Kernel always shows the worst performance, implying that Quadratic Kernel may not be a good choice in breast cancer outcome predictions.

## Discussions

**Fig. 1:**
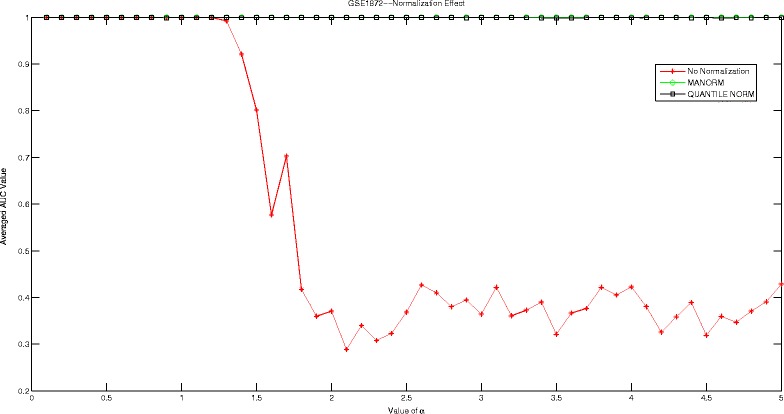
Normalization Effect on Hadamard Kernel: GSE1872

**Fig. 2:**
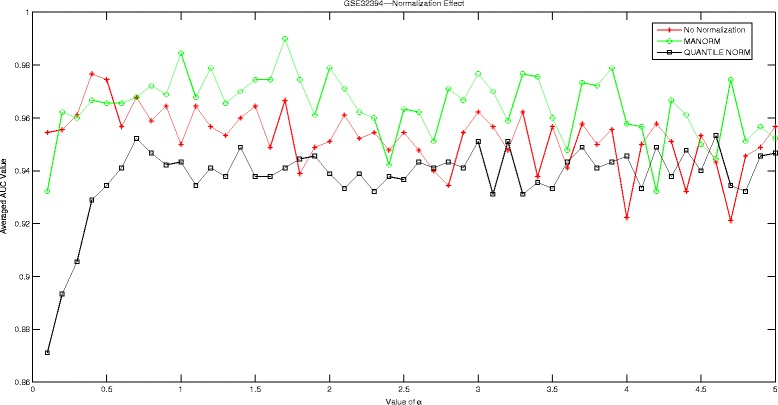
Normalization Effect on Hadamard Kernel: GSE32394

**Fig. 3:**
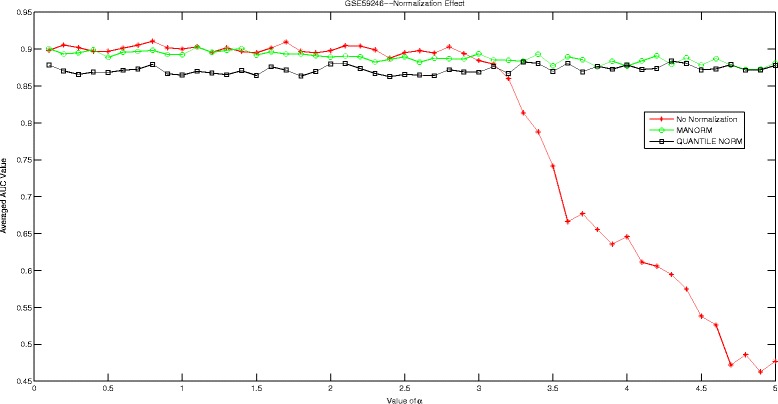
Normalization Effect on Hadamard Kernel: GSE59246

**Fig. 4:**
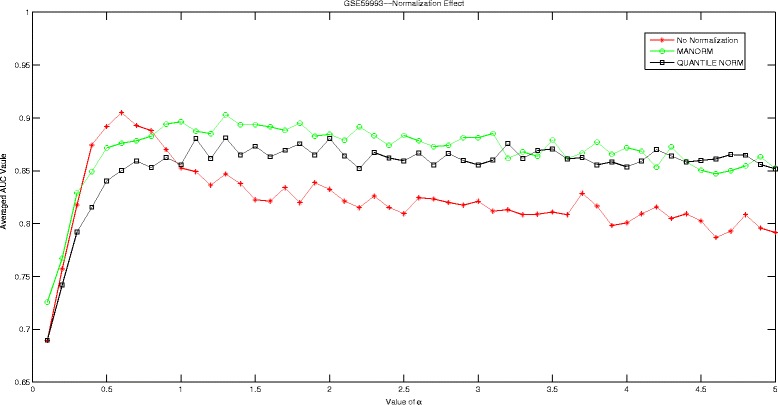
Normalization Effect on Hadamard Kernel: GSE59993

**Fig. 5:**
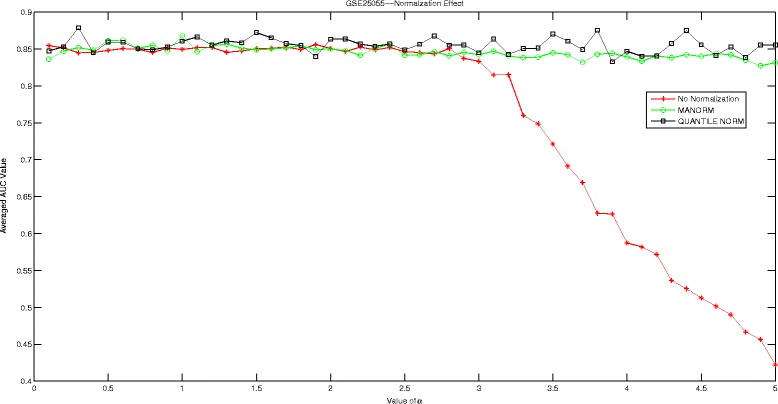
Normalization Effect on Hadamard Kernel: GSE25055

**Fig. 6:**
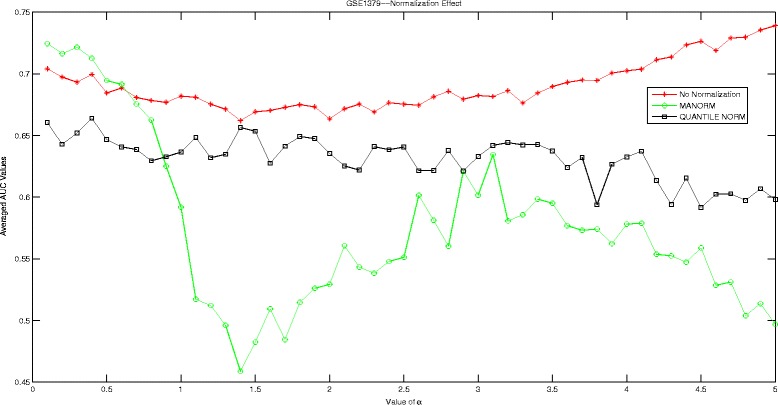
Normalization Effect on Hadamard Kernel: GSE1379

In this section, we are going to investigate the effect of normalization strategy on our proposed Hadamard kernel. As most of our datasets are microarray data, we therefore introduce ‘manorm’ which is an embedded function in Matlab for microarray data normalization. It scales the values in each column of microarray data matrix, by dividing by the mean column intensity. Besides, we also include quantile normalization for testing purpose. Quantile normalization is designed for making two distributions identical in statistical properties. We employed the AUC measured by 5-fold cross-validation run 10 times to evaluate the performance of Hadamard kernel with data normalization and without. The experimental results are recorded in supplementary files under figure names Figs. 1, 2, 3, 4, 5, and 6. It can be seen that in general Hadamard kernel after data normalization tend to perform better than the original Hadamard kernel without normalization.Green ‘ ∘’ represents ‘manorm’ normalization, red star ‘ ⋆’ represents Hadamard kernel without normalization, and black square represents ‘quantile’ normalization. For example, in Fig. 1 for GSE1872, after normalization, the performance of Hadamard kernel is approaching 100% in AUC value, while the original hadamard kernel is relatively unstable. Similar patterns can be detected for GSE32394 and GSE59993 where ‘manorm’ and ‘quantile’ normalization with Hadamard kernel perform better than Hadamard kernel without data normalization. However, some exceptions also occur where we can see that for GSE1379, Hadamard kernel without normalization tends to perform more stably. When we further check the data format for GSE1379, we can see that the data set was already normalized where the data was measured as log2 normalized ratio of Cy5/Cy3. Hence, it is not surprising. In GSE25055, the performance of Hadamard kernel without data normalization is not very stable. When *α* is relatively large, the performance decreases drastically. When normalization is done on the data, we can see that the performance becomes stable. Besides, ‘quantitle’ normalization based Hadamard kernel is slightly better than Hadamard kernel without normalization when *α*<3. In GSE59246, we can see that Hadamard kernel after normalization show comparable performance with Hadamard kernel without normalization when *α* in relatively small range. When the value of *α* increases, we can see that Hadamard kernel after normalization is more stable. To sum up, we can see that normalization positively affect the performance of Hadamard kernel.

A new perspective regards Hadamard kernel as a kernel on implicitly normalized data, hence in the following we are going to compare the performance of Hadamard kernel with other kernels under normalization. Similarly we introduce quantile normalization for testing purpose. Since correlation kernel needs to calculate the eigenspace of the correlation matrix, data after normalization sometimes yields unsolvable kernel, hence we use original correlation in this context. We still conduct 5-fold cross-validation to test the performance of different methods. In the following Table 4, we can see the comparison of Hadamard kernel on raw data and other kernel methods on normalized data. We can find that Hadamard kernel is robust as it performs best for almost all the considered datasets compared to other methods after data normalization. In GSE1872, linear kernel and quadratic kernel after normalization perform significantly better than kernels without normalization. Almost all kernels can yield 100% averaged AUC value except for RBF kernel. In GSE32394, GSE59246, GSE59993 and GSE25055, quadratic kernel after data normalization performs significantly better, competing with linear kernel. But Hadamard kernel still demonstrates the best. Normalization effect on linear kernel is demonstrated in GSE1872 and GSE1379. We can see that after normalization the performance does improves. For RBF kernel, the performance in some data sets after normalization decreases. Possible reason is that we used the optimal *σ* selected for data without normalization. When normalization is imposed, perhaps the best *σ* has changed, hence the performance in some data sets decreases.

**Table 4:** Comparison of Hadamard kernel on raw data and different methods on normalized data

Methods					
Datasets	Linear	Quadratic	RBF	Correlation	Hadamard
	kernel	kernel	kernel	kernel	kernel
GSE1872	1	1	0.2367	0.9962	1
GSE32394	0.9556	0.9556	0.8500	0.9444	**0.9833**
GSE59246	0.8546	0.8546	0.8061	0.8626	**0.8849**
GSE59993	0.8521	0.8476	0.7977	0.8277	**0.8913**
GSE25055	0.8619	0.8615	0.7914	0.7715	**0.8590**
GSE1379	0.7009	0.5017	0.7411	0.6797	**0.7623**

The bold face represents significantly best performance for different compared methods

As a generalization ability test on Hadamard kernel, we introduce some RNAseq data sets for validation. The results are illustrated in Table 5. One of the test data sets is obtained from NCBI GEO database, the accession number is GSE87517. Gene expression analyses in leukocytes sorted from normal breast tissues, ductal carcinomas in situ (DCIS), and HER2+ and triple negative invasive ductal carcinomas (IDC) were conducted. RNAseq counts are used to measure the expression levels. We have 41 samples in total, and the number of attributes is 27011. We focus on differentiating normal samples from breast tumor samples. We conduct experiments on Hadamrd kernel without data normalization and imposing quantile normalization on data for other methods. The best *σ* in RBF kernel and best *α* in Hadamard kernel are shown to be 0.01 and 0.2 respectively where details are attached in Additional file 2 (Table S1, Figure S13). We further compare on Hadamard kernel with other kernel methods through 5-fold cross-validations. Averaged AUC values are calculated as shown in Table 5. It can be seen that Hadamard kernel on raw data shows the best performance, achieving 0.9524 in AUC value. While the best performance in other kernels is achieved in Correlation kernel, yielding only 0.7189 in averaged AUC value.

**Table 5:** Comparison of Hadamard kernel on raw data and different methods on normalized data(RNA)

Methods					
Datasets	Linear	Quadratic	RBF	Correlation	Hadamard
	kernel	kernel	kernel	kernel	kernel
GSE87517	0.6022	0.4562	0.5459	0.7189	**0.9524**
GSE47462	0.7422	0.5322	0.4029	0.7506	**0.8949**
GSE48213	0.9990	0.9982	0.3375	0.9993	**0.9996**

The bold face represents significantly best performance for different compared methods

One of the test data sets is also obtained from NCBI GEO database, the accession number is GSE47462. Raw counts lncRNAs are used to measure the expression levels. We have 72 samples in total, of which 24 are normal, 25 early neoplasia, 9 carcinoma in situ, and 14 invasive cancer. The number of attributes is 2173. We focus on differentiating normal samples from breast tumor samples. The best *σ* in RBF kernel and best *α* in Hadamard kernel are shown to be 1000 and 0.5 respectively where details are attached in Additional file 2 (Table S2, Figure S14). We further compare on Hadamard kernel with other kernel methods through 10 runs 5-fold cross-validations. Averaged AUC values are calculated and the results are reported in Table 5. It can be shown that Hadamard kernel is robust and can demonstrate satisfactory performance compared to other kernels even with data normalization. The averaged AUC value in Hadamard kernel is 0.8949 while in linear kernel 0.7422. The performance in RBF kernel is not satisfactory, achieving only 0.4029 in averaged AUC value.

The third data set is under accession number GSE48213. 56 breast cancer cell lines were profiled to identify patterns of gene expression associated with subtype and response to therapeutic compounds using RNAseq technology. There are 4 unknown cell lines, with 27 samples related to Luminal, 14 samples related to Basal like breast cancer, 5 normal samples and 6 samples of Claudin-low subtype. Subtype Luminal constitutes the majority of all the considered subtypes, hence we try to differentiate Luminal from others by removing the 4 unknown samples. Hadamard kernel on raw data can yield 0.9996 in averaged AUC value. The performance in other kernels after data normalization is also comparable except in RBF kernel.

In Additional file 2: Table S4, we also record the performance of the 4 compared kernels on considered RNAseq data sets without data normalization.

In a word, we can see that Hadamard kernel is robust for dealing with expression data in general.

## Conclusions

In this paper, we proposed Hadamard Kernel for breast cancer outcome predictions. It is a valid and effective kernel for dealing with high dimensional gene expression data when they are positive valued. In particular, we have given theoretical verification on the positive semi-definiteness for all kinds of data. Through comparison with classical kernels in SVM and correlation kernel that is good at cancer predictions, we show the superiority of Hadamard Kernel. The hadamard kernel is flexible in varying the parameter *α*, the determination of optimal *α* can be devoted to our future work. We hope Hadamard kernel as a novel class of kernels can enrich kernel communities in SVM and contribute to the wider biological problems.

## Additional files

Additional file 1 Figures. Additional file 1 includes 12 figures. Figure S1 to S6 describe the performance of Hadamard kernel with different values of *α*. Figure S7 to S12 show the performance of Hadamard kernel compared with other kernel methods. (PDF 40 kb)

Additional file 2 Results on RNAseq data. Additional file 2 contains results on RNAseq data for breast cancer outcome predictions. (DOCX 157 kb)

## References

[1] DeSantis C, Siegel R, Bandi P, Jemal A (2011). Breast cancer statistics. CA Cancer J Clin.

[2] Society AC (2016). Cancer Facts & Figures.

[3] Dudoit S, Fridlyand J, Speed TP (2002). Comparison of Discrimination Methods for the Classification of Tumors Using Gene Expression Data. J Am Stat Assoc.

[4] Cox DR (2002). A Gene-Expression Signature as a Predictor of Survival in Breast Cancer. N Engl J Med.

[5] Lj V’V, Dai H, Mj VDV (2002). Gene expression profiling predicts clinical outcome of breast cancer. Nature.

[6] Vliet MHV, Reyal F, Horlings HM (2008). Pooling breast cancer datasets has a synergetic effect on classification performance and improves signature stability. BMC Genomics.

[7] Eb VDA, Verbruggen B, Heijmans BT (2016). Integrating protein-protein interaction networks with gene-gene co-expression networks improves gene signatures for classifying breast cancer metastasis. J Integr Bioinform.

[8] Maglogiannis I, Zafiropoulos E, Anagnostopoulos I (2009). An intelligent system for automated breast cancer diagnosis and prognosis using SVM based classifiers. Appl Intell.

[9] Delen D, Walker G, Kadam A (2005). Predicting breast cancer survivability: a comparison of three data mining methods. Artif Intell Med.

[10] Endo A, Shibata T, Tanaka H (2008). Comparison of Seven Algorithms to Predict Breast Cancer Survival(Contribution to 21 Century Intelligent Technologies and Bioinformatics). Biomed Fuzzy Hum Sci Off J Biomed Fuzzy Syst Assoc.

[11] Chaurasia V, Pal S (2014). Data Mining Techniques: To Predict and Resolve Breast Cancer Survivability. Int J Comput Sci Mob Comput.

[12] Aruna S, Rajagopalan DSP, Nandakishore LV (2012). Knowledge based analysis of various statistical tools in detecting breast cancer. Aust N Z J Stat.

[13] Asri H, Mousannif H, Moatassime HA (2016). Using Machine Learning Algorithms for Breast Cancer Risk Prediction and Diagnosis. Procedia Comput Sci.

[14] Jiang H, Ching WK (2012). Correlation Kernels for Support Vector Machines Classification with Applications in Cancer Data. Comput Math Meth Med.

[15] Cortes C, Cortes C, Vapnik V (1995). Support-vector networks. Mach Learn.

[16] Ajzerman MA, Braverman EM, Rozonoehr LI (1964). Theoretical foundations of the potential function method in pattern recognition learning. Autom Remote Control.

[17] Scholkopf B, Smola AJ (2001). Learning With Kernels: Support Vector Machines, Regularization, Optimization, and Beyond.

[18] Bapat RB (1988). Multinomial probabilities, permanents and a conjecture of Karlin and Rinott. Proc Am Math Soc.

[19] Reams R (1999). Hadamard inverses, square roots and products of almost semidefinite matrices. Linear Algebra Appl.

[20] Breast Cancer Data. http://www.ncbi.nlm.nih.gov/. Accessed 6 May 2017.

[21] Sgroi DC, Haber DA, Ryan PD (2004). RE: A two-gene expression ratio predicts clinical outcome in breast cancer patients treated with tamoxifen. Cancer Cell.

[22] Hanley JA, Mcneil BJ (2008). A method of comparing the areas under receiver operating characteristic curves derived from the same cases. Radiology.

[23] Mamitsuka H (2006). Selecting features in microarray classification using ROC curves. Pattern Recog.

[24] Flach PA, Hernândez-Orallo J, Ramirez CF. A Coherent Interpretation of AUC as a Measure of Aggregated Classification Performance. International Conference on Machine Learning, ICML, 2011. Bellevue, Washington, USA, June 28-July.DBLP; 2011. pp. 657–64.

