# Supplementary material for: Hadamard Kernel SVM with applications for breast cancer outcome predictions
Source: BMC Syst Biol. 2017 Dec 21;11(Suppl 7):138. doi: 10.1186/s12918-017-0514-1 (PMC5763304; doi:10.1186/s12918-017-0514-1)
Supplement: Supplementary file 1 — Figures. Additional file 1 includes 12 figures. Figure S1 to S6 describe the performance of Hadamard kernel with different values of α. Figure S7 to S12 show the performance of Hadamard kernel compared with other kernel methods. (PDF 40 kb) [file 12918_2017_514_MOESM1_ESM.pdf]

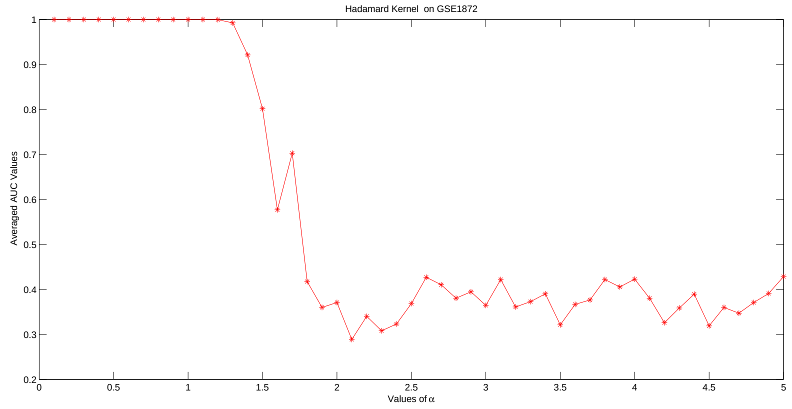

Fig S 1. AUC Values for Hadamard Kernel: GSE1872

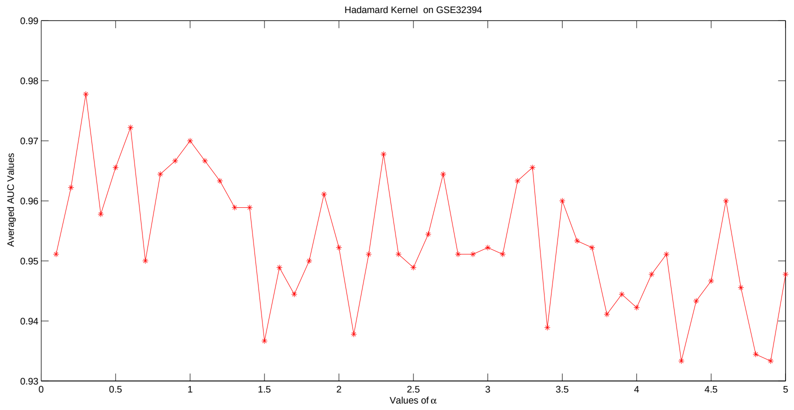

Fig S 2. AUC Values for Hadamard Kernel: GSE32394

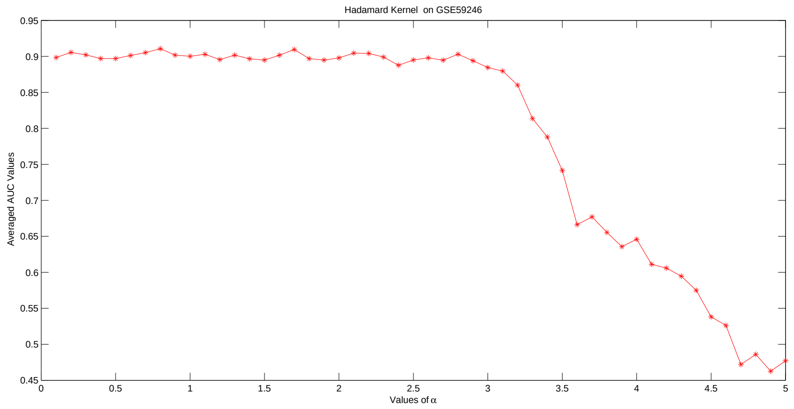

Fig S 3. AUC Values for Hadamard Kernel: GSE59246

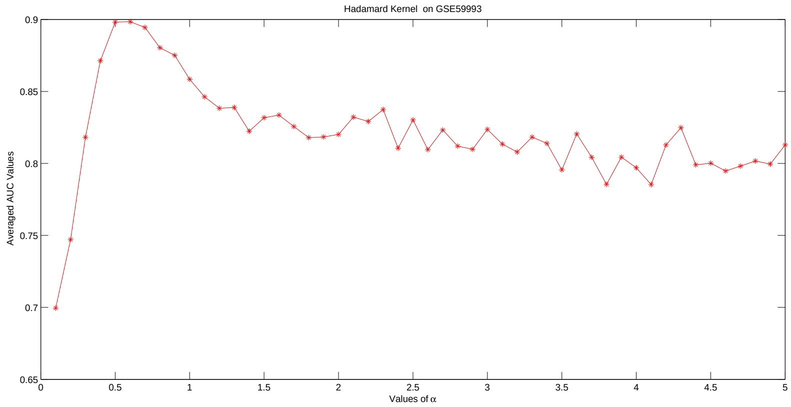

Fig S 4. AUC Values for Hadamard Kernel: GSE59993

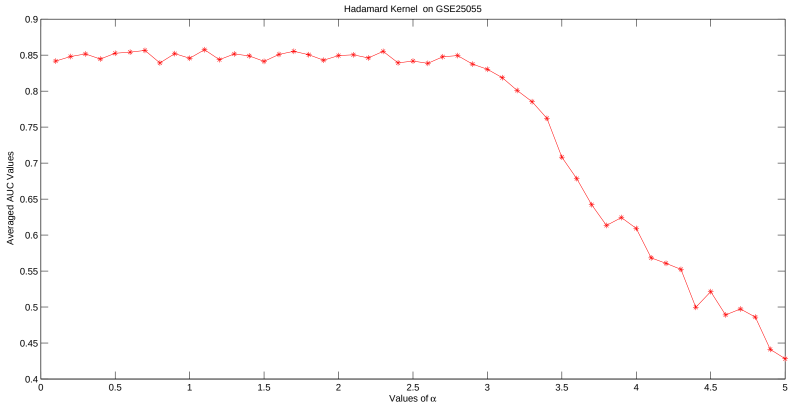

Fig S 5. AUC Values for Hadamard Kernel: GSE25055

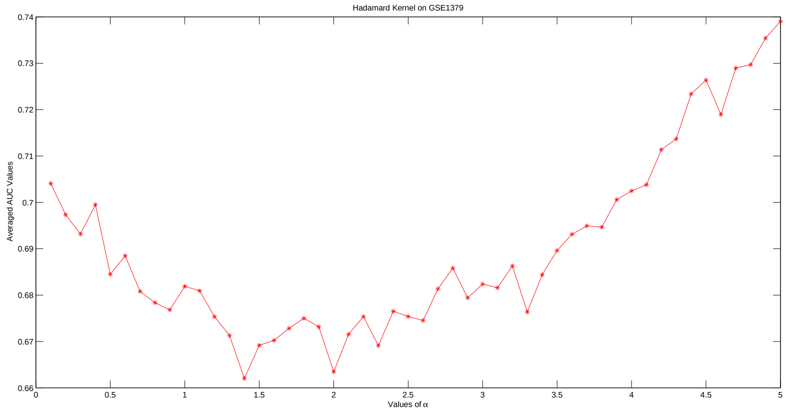

Fig S 6. AUC Values for Hadamard Kernel: GSE1379

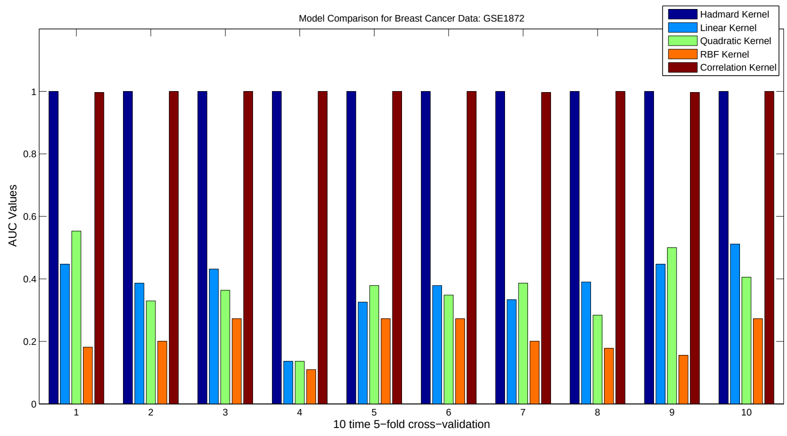

Fig S 7. AUC Values for Comparison of Hadamard Kernel and other kernels: GSE1872

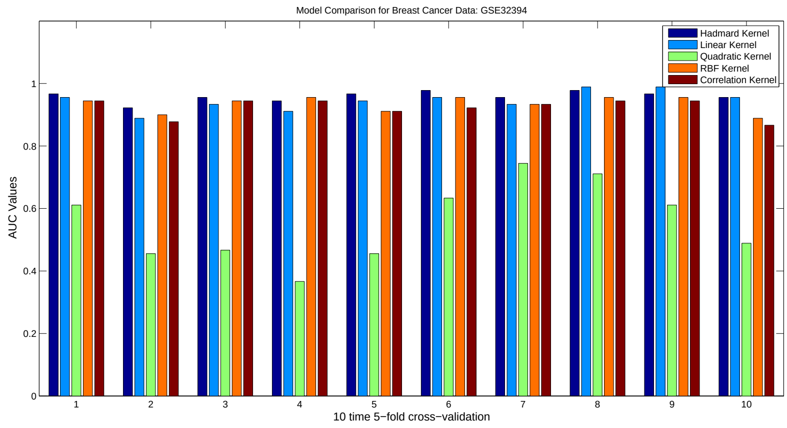

Fig S 8. AUC Values for Comparison of Hadamard Kernel and other kernels: GSE32394

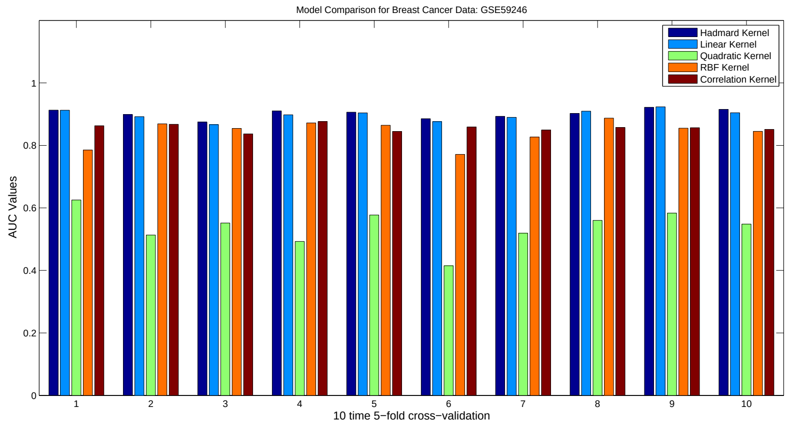

Fig S 9. AUC Values for Comparison of Hadamard Kernel and other kernels: GSE59246

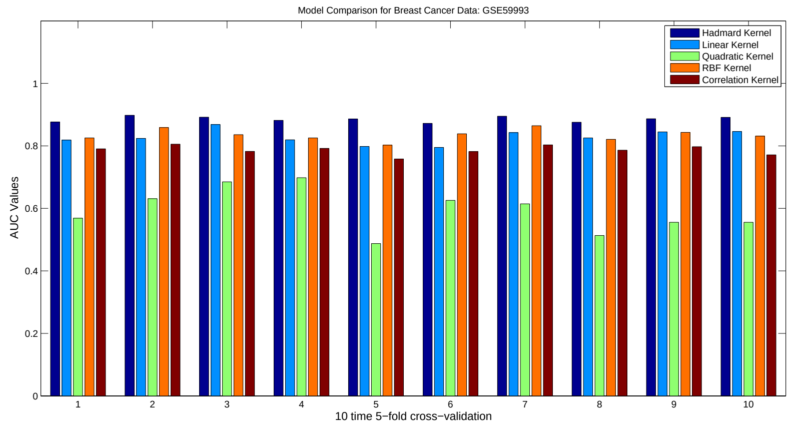

Fig S 10. AUC Values for Comparison of Hadamard Kernel and other kernels: GSE59993

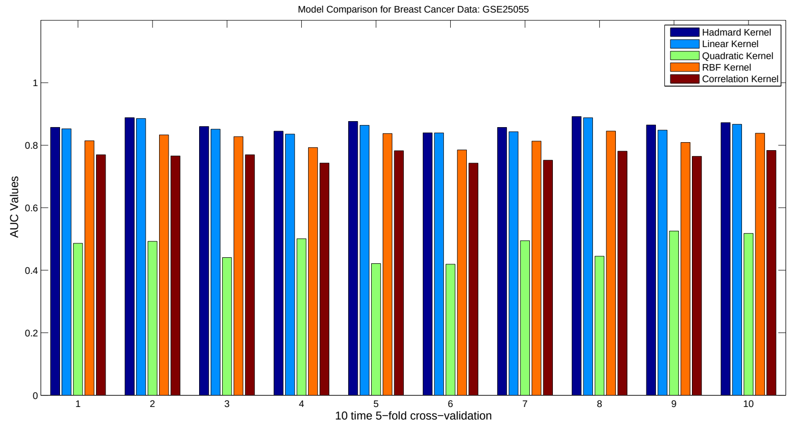

Fig S 11. AUC Values for Comparison of Hadamard Kernel and other kernels: GSE25055

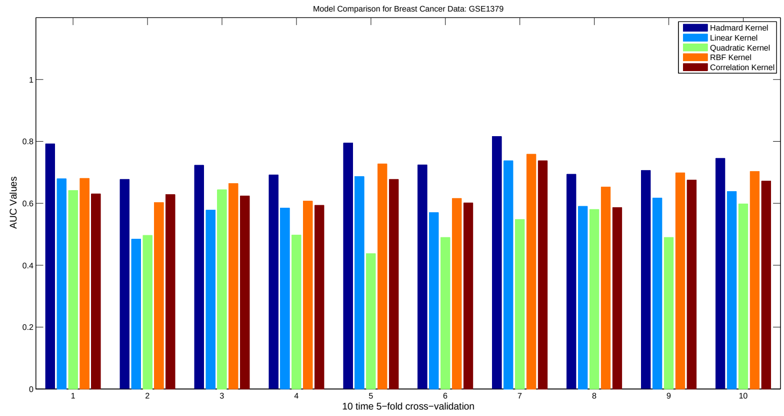

Fig S 12. AUC Values for Comparison of Hadamard Kernel and other kernels: GSE1379
