# Supplementary material for: Hadamard Kernel SVM with applications for breast cancer outcome predictions
Source: BMC Syst Biol. 2017 Dec 21;11(Suppl 7):138. doi: 10.1186/s12918-017-0514-1 (PMC5763304; doi:10.1186/s12918-017-0514-1)
Supplement: Supplementary file 2 — Results on RNAseq data. Additional file 2 contains results on RNAseq data for breast cancer outcome predictions. (DOCX 157 kb) [file 12918_2017_514_MOESM2_ESM.docx]

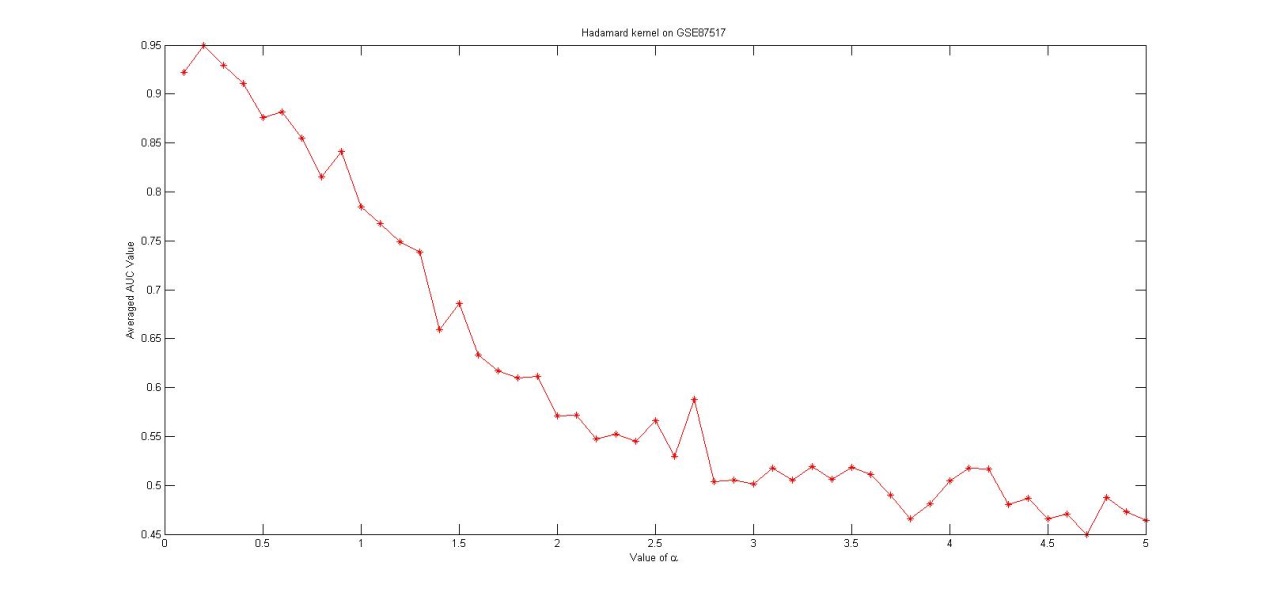


FigS13 Hadamard kernel on GSE87517 for different values of alpha(Best alpha=0.2)


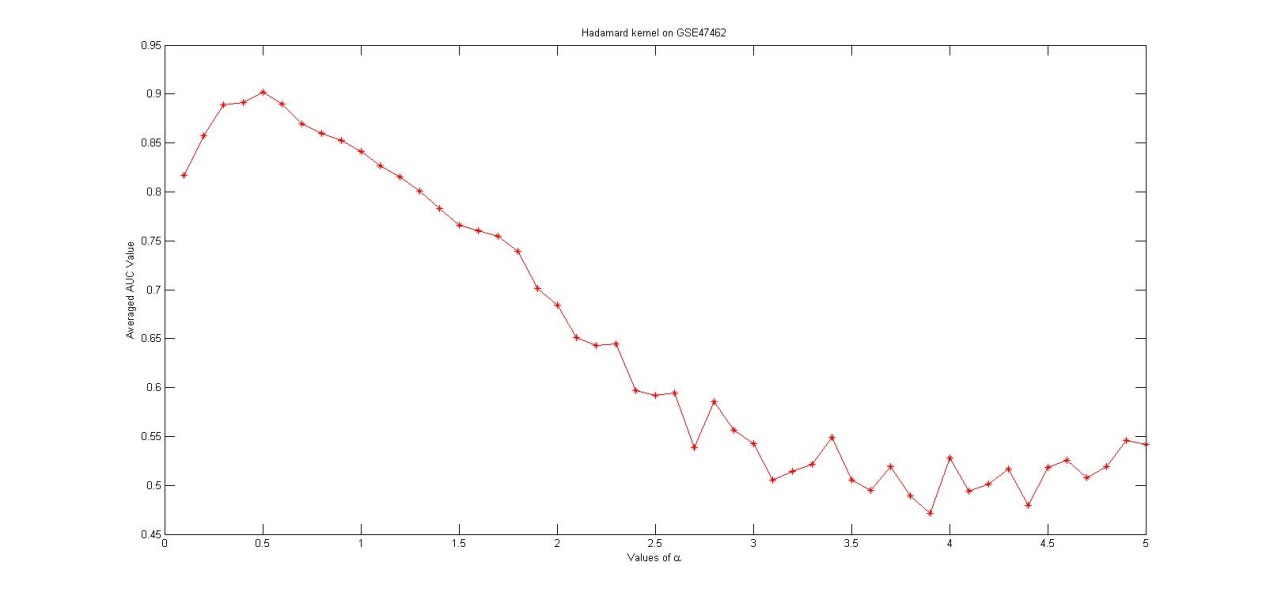


FigS14 Hadamard kernel on GSE47462 for different values of alpha(Best alpha=0.5)


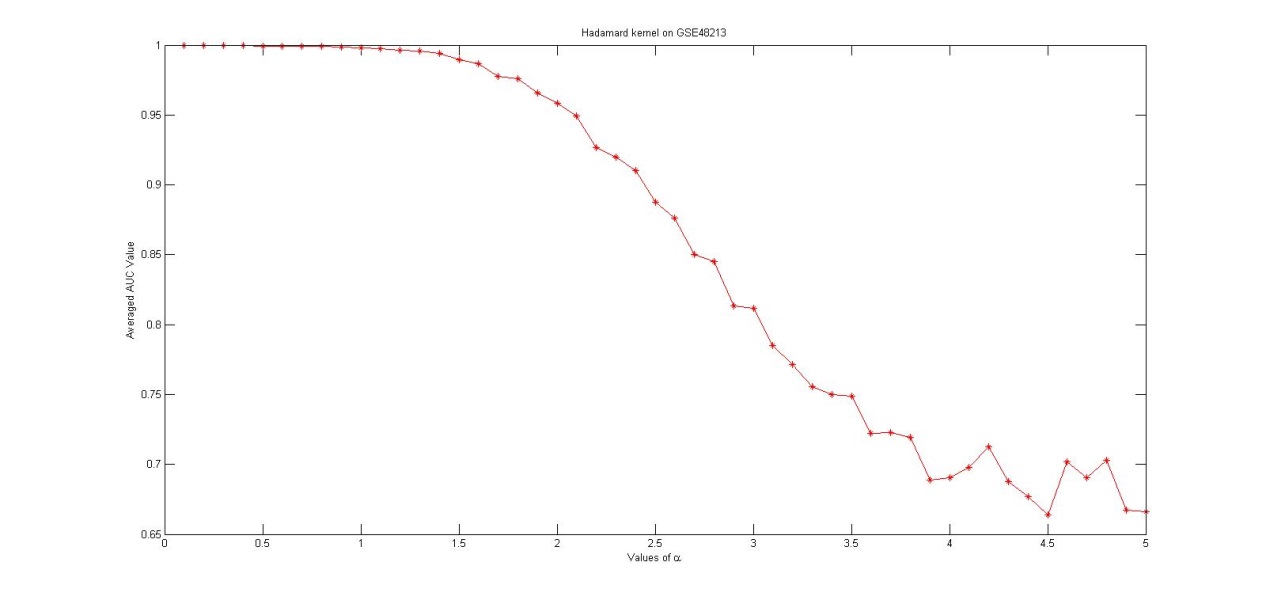


FigS15 Hadamard kernel on GSE48213 for different values of alpha(Best alpha=0.3)

Table S1 Sigma selection for RBF kernel(GSE87517)

| 0.409091 | 0.399522 | 0.497608 | 0.423445 | 0.342105 | 0.344498 | 0.349282 | 0.342105 | 0.480861 | 0.437799 |  | sigma=10^3 |
| --- | --- | --- | --- | --- | --- | --- | --- | --- | --- | --- | --- |
| 0.409091 | 0.399522 | 0.497608 | 0.423445 | 0.342105 | 0.344498 | 0.349282 | 0.342105 | 0.480861 | 0.437799 |  | sigma=10^2 |
| 0.409091 | 0.399522 | 0.497608 | 0.423445 | 0.342105 | 0.344498 | 0.349282 | 0.342105 | 0.480861 | 0.437799 |  | sigma=10 |
| 0.409091 | 0.399522 | 0.497608 | 0.423445 | 0.342105 | 0.344498 | 0.349282 | 0.342105 | 0.480861 | 0.437799 |  | sigma=1 |
| 0.409091 | 0.399522 | 0.497608 | 0.423445 | 0.342105 | 0.344498 | 0.349282 | 0.342105 | 0.480861 | 0.437799 |  | sigma=0.1 |
| 0.478469 | 0.746411 | 0.684211 | 0.423445 | 0.566986 | 0.344498 | 0.602871 | 0.375598 | 0.557416 | 0.669856 |  | sigma=0.01 |
| 1st AUC | 2nd AUC | 3rd AUC | 4th AUC | 5th AUC | 6th AUC | 7th AUC | 8th AUC | 9th AUC | 10th AUC |  | GSE87517 |

Table S2 Sigma selection for RBF kernel(GSE47462)

| 0.313368 | 0.436632 | 0.434896 | 0.414931 | 0.327257 | 0.361111 | 0.486111 | 0.386285 | 0.326389 | 0.337674 |  | sigma=10^3 |
| --- | --- | --- | --- | --- | --- | --- | --- | --- | --- | --- | --- |
| 0.304688 | 0.387153 | 0.391493 | 0.330729 | 0.354167 | 0.318576 | 0.457465 | 0.375868 | 0.297743 | 0.368056 |  | sigma=10^2 |
| 0.304688 | 0.387153 | 0.391493 | 0.330729 | 0.354167 | 0.318576 | 0.457465 | 0.375868 | 0.297743 | 0.368056 |  | sigma=10 |
| 0.304688 | 0.387153 | 0.391493 | 0.330729 | 0.354167 | 0.318576 | 0.457465 | 0.375868 | 0.297743 | 0.368056 |  | sigma=1 |
| 0.304688 | 0.387153 | 0.391493 | 0.330729 | 0.354167 | 0.318576 | 0.457465 | 0.375868 | 0.297743 | 0.368056 |  | sigma=0.1 |
| 0.304688 | 0.387153 | 0.391493 | 0.330729 | 0.354167 | 0.318576 | 0.457465 | 0.375868 | 0.297743 | 0.368056 |  | sigma=0.01 |
| 1st AUC | 2nd AUC | 3rd AUC | 4th AUC | 5th AUC | 6th AUC | 7th AUC | 8th AUC | 9th AUC | 10th AUC |  | GSE47462 |

Table S3 Sigma selection for RBF kernel(GSE48213)

| 0.445926 | 0.365926 | 0.404444 | 0.343704 | 0.37037 | 0.32 | 0.417778 | 0.34963 | 0.425185 | 0.288889 |  | sigma=10^3 |
| --- | --- | --- | --- | --- | --- | --- | --- | --- | --- | --- | --- |
| 0.411852 | 0.346667 | 0.38963 | 0.328889 | 0.355556 | 0.32 | 0.4 | 0.34963 | 0.408889 | 0.277037 |  | sigma=10^2 |
| 0.411852 | 0.346667 | 0.38963 | 0.328889 | 0.355556 | 0.32 | 0.4 | 0.34963 | 0.408889 | 0.277037 |  | sigma=10 |
| 0.411852 | 0.346667 | 0.38963 | 0.328889 | 0.355556 | 0.32 | 0.4 | 0.34963 | 0.408889 | 0.277037 |  | sigma=1 |
| 0.411852 | 0.346667 | 0.38963 | 0.328889 | 0.355556 | 0.32 | 0.4 | 0.34963 | 0.408889 | 0.277037 |  | sigma=0.1 |
| 0.420741 | 0.346667 | 0.404444 | 0.337778 | 0.355556 | 0.32 | 0.408889 | 0.358519 | 0.423704 | 0.277037 |  | sigma=0.01 |
| 1st AUC | 2nd AUC | 3rd AUC | 4th AUC | 5th AUC | 6th AUC | 7th AUC | 8th AUC | 9th AUC | 10th AUC |  |  |

Table S4. Comparison of Hadamard kernel on raw data(-) and different methods on normalized data(+)

| Datasets | Linear | Quadratic | RBF | Correlation | Hadamard |
| --- | --- | --- | --- | --- | --- |
| GSE87517 | 0.6022(+) | 0.4562(+) | 0.5459(+) | 0.7189(+) | 0.9524(-) |
|  | 0.5909(-) | 0.4830(-) | 0.4730(-) | 0.6964(-) | 0.9447(-) |
| GSE47462 | 0.7422(+) | 0.5322(+) | 0.4029(+) | 0.7506(+) | 0.8949(-) |
|  | 0.7368(-) | 0.4829(-) | 0.3878(-) | 0.7351(-) | 0.8986(-) |
| GSE48213 | 0.9990(+) | 0.9982(+) | 0.3375(+) | 0.9993(+) | 0.9996(-) |
|  | 0.9670(-) | 0.9681(-) | 0.3231(-) | 0.9471(-) | 0.9982(-) |

Values are measured as averaged AUC values. (+) represents data normalization, (-) represents raw data.
